# Reparative effects of *Schizophyllum commune* oat bran fermentation broth on UVB-induced skin inflammation *via* the JAK/STAT pathway

**DOI:** 10.1186/s40643-024-00792-2

**Published:** 2024-07-25

**Authors:** Zixin Song, Jiman Geng, Dongdong Wang, Jiaxuan Fang, Ziwen Wang, Changtao Wang, Meng Li

**Affiliations:** 1 College of Light Industry Science and Engineering, Beijing Technology & Business University, 11 Fucheng Road, Haidian District, Beijing, 100048 China; 2 Beijing Key Laboratory of Plant Resource Research and Development, Beijing Technology and Business University, 11 Fucheng Road, Beijing, 100048 China

**Keywords:** Oat bran, *Schizophyllum commune*, Skin inflammation, JAK/STAT pathway

## Abstract

Human immortal keratinocyte cells (HaCaT) are induced with UVB to establish an injury model. This model is utilized to investigate whether oat bran fermentation broth (OBF) has a reparative effect on skin inflammation and damage to the skin barrier caused by UVB irradiation. The results show that compared with unfermented oat bran (OB), OBF exhibits higher structural homogeneity, increased molecular weight size, active substances content, and in vitro antioxidant activity. OBF has a scavenging effect on excess reactive oxygen species (ROS) and increases the intracellular levels of antioxidant enzymes. It was found that OBF has a stronger inhibitory effect on the release of inflammatory factors than OB. It increases the synthesis of AQP3 and FLG proteins while decreasing the secretion of KLK-7. OBF can inhibit the transcription level of inflammatory factors by suppressing the JAK/STAT signaling pathway. Safety experiments demonstrate that OBF has a high safety profile.

**Graphical Abstract:**

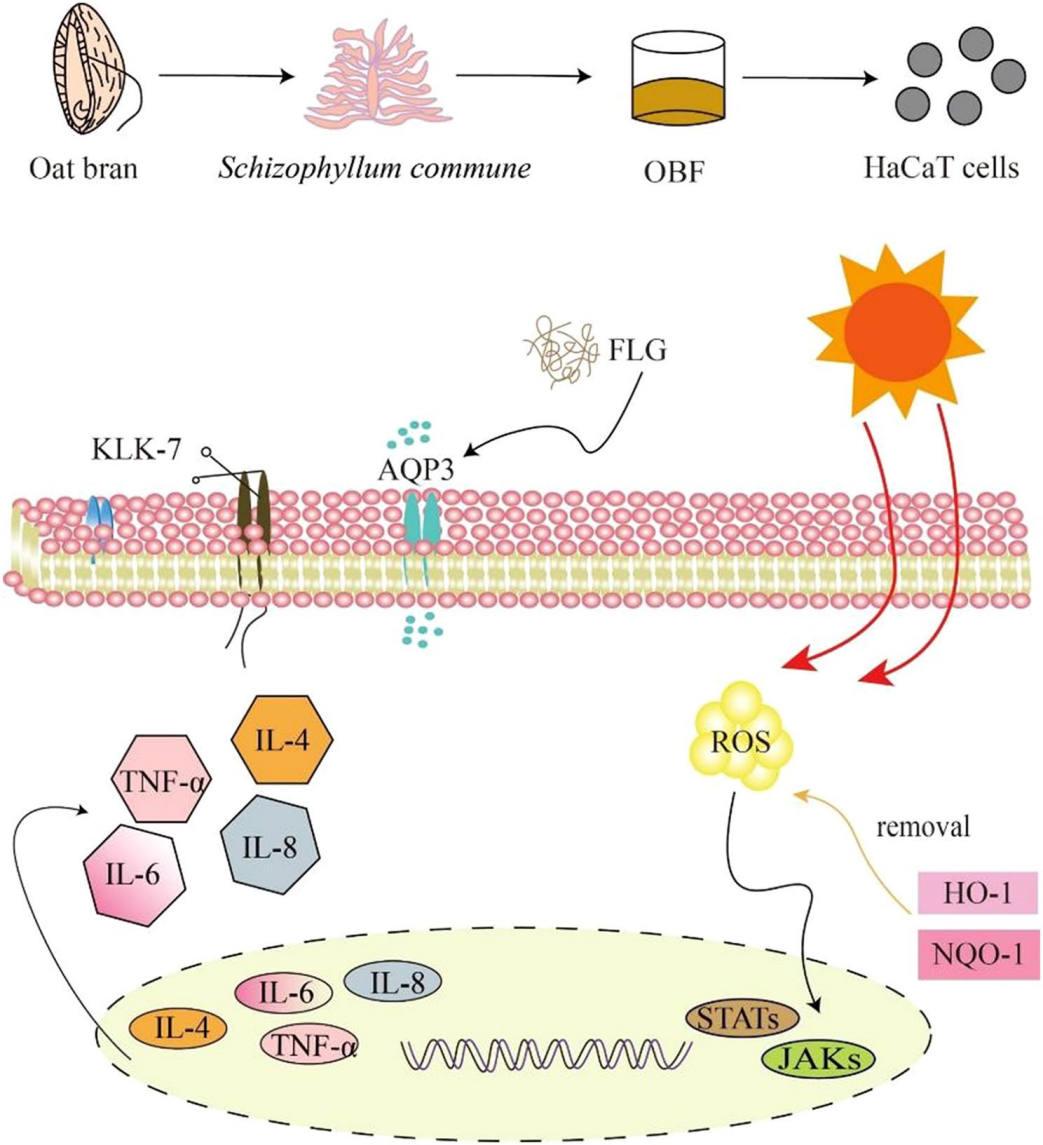

## Introduction

Oat (*Avena sativa L.*) is an ancient crop that is widely grown around the world and has been cultivated for more than 2,000 years (Wang et al. 2022). The processing of oats produces a large number of by-products such as oat bran. Oat bran contains various chemical substances such as dietary fiber, vitamins, proteins, polysaccharides, phenolic compounds and other active ingredients (Khider et al. 2022), which contribute to its numerous physiological and pharmacological activities. These activities include anti-inflammatory and antioxidant properties, protection of liver health, and reduction of the risk of cardiovascular disease, among others (Zhang et al. 2023). Due to its good nutritional function and high safety, oat bran is currently widely used in the food, medicine and cosmetics industries (Singh et al. 2013).

The polyphenols contained in oats are mainly found in the outer layer of the kernel, usually in a bound state, and it has been shown that the content of polyphenolic compounds in oat bran is closely related to its antioxidant capacity. The primary ingredient in oat bran is oat β-glucan, which can be utilized to reduce cholesterol levels, regulate glucose absorption, and prevent cardiovascular diseases (Sun et al. 2020). Liu extracted polysaccharides from crude oat bran and ultrafine ground oat bran using the alcohol precipitation method, and compared the in vitro antioxidant capacity of the two polysaccharides through DPPH radical scavenging experiment, FRAP iron ion reduction ability experiment, and ABTS radical scavenging experiment. The experimental results showed that polysaccharides extracted from ultrafine ground oat bran exhibited superior in vitro antioxidant activity (Liu et al. 2016). Between 65% and 90% of the total phosphorus in oat bran is stored as inositol hexakisphosphate. The high content of inositol hexakisphosphate is a major factor limiting the efficient utilization of oat bran. However, the fermentation process can degrade inositol hexakisphosphate, facilitating the high-value utilization of oat bran (Kumar et al. 2021).

*Schizophyllum commune* (*S. commune*) is a fungus of Schizophyllaceae and Schizophyllum, also known as white ginseng, chicken hair fungus and white flower. It is widely distributed, can inhabit dead logs and usually grows abundantly in the rainy season (Almasi et al. 2019). *S. commune* is a kind of edible and medicinal fungus that contains a variety of biologically active components such as polyphenolic compounds, proteins, polysaccharides, and terpenoids (Abd Razak et al. 2024). It is a multifunctional fungal cell factory which can produce various metabolites such as hydrolase, bioethanol and biosurfactant, as well as cellulase, pectinase and other enzymes to carry out the hydrolysis of cellulose, hemicelluloses and lignin (Debnath et al. 2022). The fermented grains of *S. commune* can effectively reduce cellular oxidative stress damage caused by UVA irradiation and H_2_O_2_ induction through the PI3K-AKT signaling pathway (Cheng et al. 2023).

The JAK/STAT pathway is essential for intracellular cytokine signaling facilitated by Type I and II cytokine receptors (Chapman and Kwa 2022). It involves two protein families: Janus protein tyrosine kinases (JAKs) and signal transducers and activators of transcription proteins (STATs) (Tu et al. 2011). JAK is an intracellular upstream signaling pathway. When it is activated and phosphorylated, STATs are activated, and STAT monomers dissociate from docking sites, dimerize and translocate to the nucleus where they interact with specific DNA-binding elements to regulate multiple target genes (Masullo et al. 2014). Suppressor of cytokine signaling (SOCS) molecules are a class of endogenous cytokine-dependent signaling inhibitors. In order to limit inflammatory cytokine overstimulation, keratinocytes express SOCS molecules. At the molecular level, SOCS1 and SOCS3 can act as pseudo-substrates exerting an inhibitory effect on JAK1 and JAK2, which in turn prevents the activation of STATs (Morelli et al. 2018). It has been shown that ROS is an effective inducer of the JAK-STAT signaling pathway activation. Aloe-emodin can inhibit the activation of the JAK1-STAT1/3 pathway by scavenging excessive ROS, thereby inhibiting the nuclear translocation of STAT1/3 in RAW 264.7 cells and alleviating the inflammatory response caused by LPS induction (Wang et al. 2020).

In general, a dynamic equilibrium is maintained between the production and removal of reactive oxygen species (ROS) in the body. However, when exposed to large amounts of UVB irradiation for an extended period, excessive ROS are generated. This not only leads to the peroxidation of macromolecules in the cells and induces severe oxidative damage but may also activate apoptosis-related signaling pathways, promoting programmed cell death (Liu et al. 2018). In addition, excessive UVB exposure induces the production of inflammatory intermediates, which in turn, leads to skin inflammation (Martinez et al. 2015). When the skin is in an inflammatory state, its barrier function is disrupted, making it easier for harmful substances to penetrate and exacerbating skin inflammation.

**Fig. 1:**
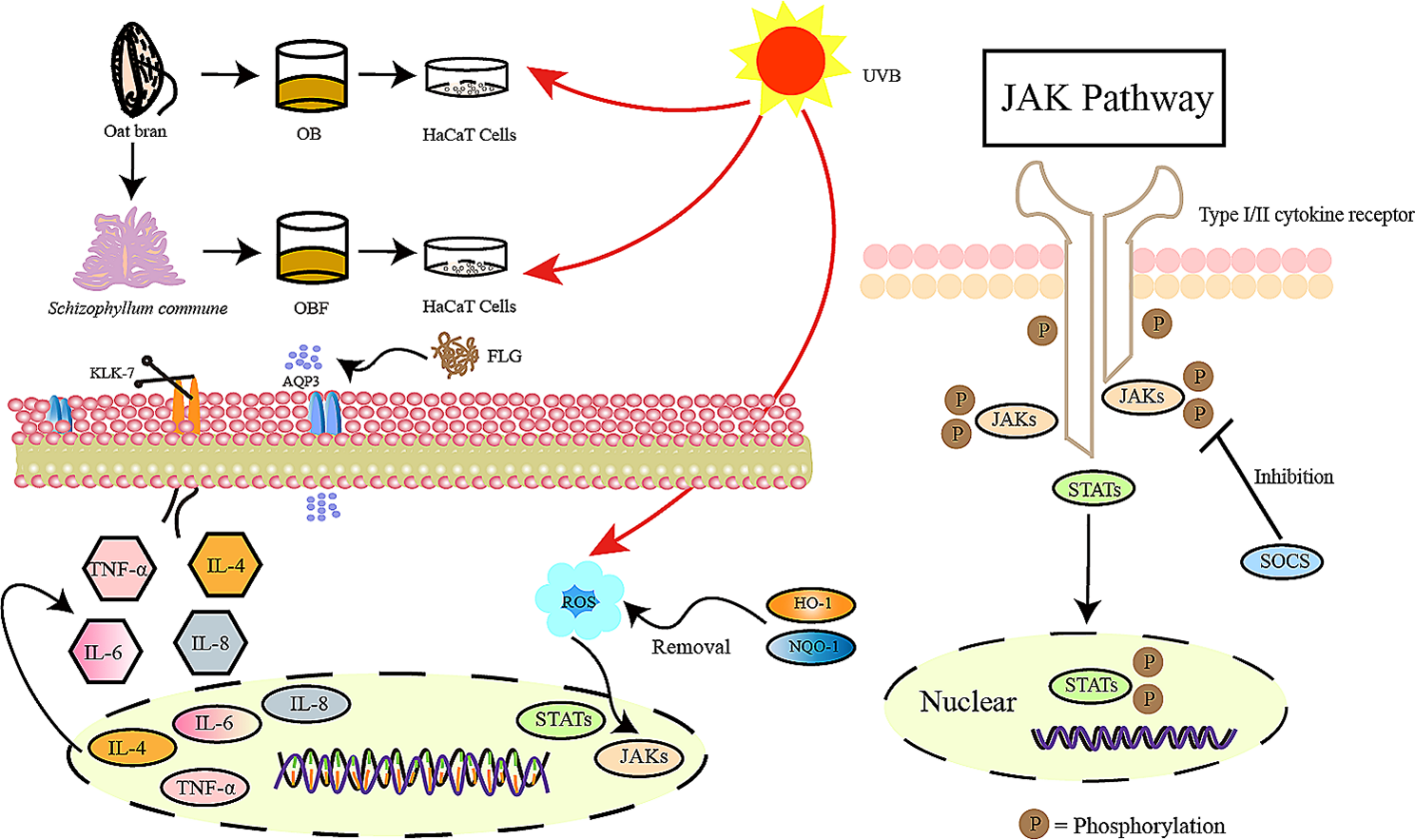
Reparative effects of OB and OBF on HaCaT cells damaged by UVB

In this study, as shown in Fig. 1, we chose to utilize *S. commune* to ferment oat bran (OBF). First, the study examined the impact of *S. commune* fermentation on the physical properties, active substance content, and in vitro antioxidant capacity of oat bran. Subsequently, we established a cell injury model by exposing HaCaT cells to UVB irradiation. We assessed cell viability using the CCK8 assay, examined cell migration ability at specific sample concentrations, and evaluated the impact of the samples on cellular oxidative stress, inflammatory response, and barrier function. This analysis enabled us to investigate whether OBF exhibits reparative effects on UVB-induced inflammation and barrier damage. Finally, we conducted experiments at the molecular level to examine the impact of the JAK/STAT pathway on the reparative properties of OBF in UVB-induced skin inflammation. Subsequently, we evaluated the safety of OBF through the chicken embryo chorioallantoic membrane test and red blood cell hemolysis test.

## Materials and methods

### Materials

*Schizophyllum commune* (strain number: CGMCC40388, Center for General Microbiology, China Microbial Strain Preservation and Management Committee, China); Human immortal keratinocyte (HaCaT) cells (Cell Resource Center of Peking Union Medical College); sodium carbonate, rutin standard, sodium nitrite, aluminum nitrate, anhydrous ethanol and copper sulfate (Sinopharm Group); Cell Counting Kit-8 (Biorigin); DMEM medium and pancreatin (GIBCO Life Technologies); ROS detection kits (Beyotime); qRT-PCR instrument (QuanStudio 3, Thermoscientific).

### Preparation of OBF

Oat bran was crushed and passed through a 100 mesh sieve. 300 mL of deionized water and 3 g of oat bran were mixed evenly, sterilized in a 121℃ autoclave for 20 min and cooled to room temperature (rt). *S. commune* solution was added for fermentation, and the mixture was cultured in a 28℃ constant temperature incubator for 48 h. It was rotated on a shaking table at 180 r/min, cooled to room temperature, and then centrifuged at 4800 r/min for 25 min. The supernatant was then taken to obtain OBF. Oat bran unfermented broth (OB) was obtained according to the same pretreatment method as OBF without the addition of *S. commune.*

### GPC and SEM

The molecular weight sizes of OBF and OB were determined by the gel permeation chromatography-laser light scattering in line (GPC-LS-IR) method, and the morphological characteristics of the two samples, OBF and OB, were determined using an FEI Nova Nano SEM 450 instrument.

The experiments were conducted using an RID-20 oscillometric refractive detector (Shimadzu, Japan) and a TSKgel GMPWXL aqueous gel chromatography column (TOSOH, Japan). The mobile phase for the GPC consisted of a 0.1 M NaNO_3_ + 0.05% NaN_3_ aqueous solution with a flow rate of 0.6 mL/min and a column temperature of 35℃.

A small amount of powder was placed on the sample stage using conductive tape and then secured in place. Subsequently, gold was sprayed onto the sample for vacuum testing. The morphological characteristics of the samples were observed using a scanning electron microscope with an accelerating voltage of 3.0 kV, a working distance of 3.8 mm, and magnifications of 100, 200, 500 and 1000 times sequentially.

### Determination of OBF active substance content

#### Total phenols

The content of total phenolic compounds in OBF was determined by the Folin-Phenol method (Fang et al. 2023). Gallic acid was used as the standard, and various concentrations of gallic acid standard solutions were prepared to create the standard curve.

#### Total sugars

A Total Sugar Content Detection Kit (Suolaibao Biotech Co., Ltd., BC2710) was used to detect the total sugar content in the samples. Various concentrations of glucose solutions were prepared for the standard curve. Specific experimental operations were performed according to the instructions.

#### Total proteins

A BCA Protein Content Assay Kit (Biorigin, BN27109) was used to measure the total proteins content in OBF and OB. The protein standards provided in the kit were diluted to various concentrations to create the standard curve. Specific experimental operations were performed according to the instructions.

### Determination of OBF in vitro antioxidant activity

#### DPPH free radical scavenging experiment

The specific method for DPPH radical scavenging experiments were conducted following the procedures outlined by Zhao (Zhao et al. 2023). DPPH was accurately weighed 8 mg and diluted in 100 mL of anhydrous ethanol. Set up the sample tube (A_1_), the control tube (A_2_), and the sample blank tube (A_3_). Add 1 mL of different concentrations of the sample and 1 mL of DPPH to tube A_1_, 1 mL of distilled water and 1 mL of DPPH to tube A_2_, and 1 mL of different concentrations of the sample and 1 mL of distilled water to tube A_3_. The solution was mixed well and the OD517 was measured after 30 min of reaction protected from light.

1 $$\begin{aligned}\text{Scavenging capacity of samples for DPPH radicals}\%&=[(\text{A}_{2}-\text{A}_{1}+\text{A}_{3})\\&\;/\text{A}_{2}]\times 100\%\end{aligned}$$

#### Hydroxyl radical scavenging experiment

Hydroxyl radical scavenging experiments were performed with reference to Zhang (Zhang et al. 2022). Experimental methods were adjusted appropriately. First, a 8.0 mmol/L of FeSO_4_ solution, a 3.0 mmol/L of salicylic acid solution, and a 0.02 mmol/L of H_2_O_2_ solution were prepared. Set up the blank tube (A_0_), the sample tube (A_1_), and the sample background tube (A_2_). Add 0.3 mL of FeSO_4_ to tubes A_0_, A_1_, and A_2_, 1 mL of salicylic acid solution to tubes A_0_ and A_1_, 1 mL of various concentrations of the sample to both A_1_ and A_2_, 1.45 mL of distilled water to tubes A_0_ and A_2_, and 0.45 mL of distilled water to tube A_1_. Finally, add 0.25 mL of H_2_O_2_ solution to all three sets of tubes. The reaction was carried out in a water bath at a temperature of 37℃ for 1 h, the tubes were determined the OD510.

2 $$\begin{aligned}\begin{array}{l}{\text{Scavenging capacity of samples}}\\{\text{for Hydroxyl radicals}\%}\end{array}&=[\text{A}_{1}-(\text{A}_{2}-\text{A}_{3})\\&\,/\text{A}_{1}]\times 100\%\end{aligned}$$

#### Total antioxidant capacity determination

The total antioxidant capacity of both samples was determined using total antioxidant capacity assay kits, specifically the ABTS method and the FRAP method (Beyotime, China).

The ABTS working masterbatch was initially prepared and left at room temperature, shielded from light, for 12–16 h. Subsequently, after dilution with PBS, the absorbance value at 734 nm was measured to be 0.7 ± 0.05 in order to obtain the ABTS working solution. The 10 mM Trolox standard solution was diluted to 0.15, 0.3, 0.6, 0.9, 1.2, and 1.5 mM with distilled water and used to create the standard curve. Add 200 µL of ABTS working solution to each assay well in a 96-well plate. Blank control wells, standard curve detection wells, and sample detection wells were set up. Subsequently, 10 µL of distilled water, 10 µL of various concentrations of standard solutions, and 10 µL of samples were added sequentially and thoroughly mixed. The reaction was conducted at room temperature for 3 min, and the absorbance value at 734 nm was measured. The total antioxidant capacity of the samples was calculated based on the plotted standard curve.

FRAP working solution was prepared and incubated at 37℃. Dilute a 100 mM FeSO_4_ solution to concentrations of 0.15, 0.3, 0.6, 0.9, 1.2, and 1.5 mM with distilled water for the standard curve. 180 µL of FRAP working solution was added to each assay well of the 96-well plate. Blank control wells, standard curve assay wells, and sample assay wells were set up. Sequentially, 5 µL of distilled water, 5 µL of different concentrations of FeSO_4_ standard solution, and 5 µL of samples were added. The total antioxidant capacity of the samples was calculated based on the standard curve. The 96-well plate was incubated at 37℃ for 3 min. The absorbance value at 593 nm was determined, and the total antioxidant capacity of the samples was calculated using the standard curve.

### Cell culture and viability determination

The effect of the samples on cell viability was tested with reference to Zhang (Zhang et al. 2022). DMEM serum medium was prepared using fetal bovine serum (10%) and penicillin-streptomycin (1%). HaCaT cell suspensions were added in 96-well plates (Thermoscientific) at 100 µL volume per well (8 × 10^3^-1.2 × 10^4^ cells). The acellular group does not lay cells, but directly adds serum-containing culture medium. UVB damage modeling of HaCaT cells was established using UV crosslinker. The 96-well plates were then exposed to UVB irradiation conditions of 20 mJ/cm^2^ for 40 s. The control group did not undergo UVB irradiation. The plates were washed twice with PBS. Then, 100 µL of serum-free medium was added to groups C and M, and various concentrations of samples were added to the sample group. The plates were then placed in the incubator for 24 h. The CCK8 method was used to study the cytotoxicity of OBF and its role in repairing UVB damage. 100 µL of serum-free medium and 10 µL of CCK8 solution were added to each well of a 96-well plate for the assay, followed by an incubation in the incubator for 2–4 h. The OD450 was measured using an enzyme labeling instrument.

3 $$\begin{aligned} \text{Cell viability}&= (\text{sample group OD}-\text{acellular group OD})\\&\;/(\text{control group OD}-\text{acellular group OD})\times 100\% \end{aligned}$$

### Cell migration capacity assay

In a 6-well plate, 7 × 10^5^-8 × 10^5^ cells were spread in each well, 2 mL of serum medium was added and the plates were incubated in an incubator for 24 h. HaCaT cells growing adherent to the wall at the bottom of the 6-well plate were scratched with the tip of the 200 µL pipette gun. After washing the plate, 1 mL of PBS was added and pictures were taken with a fluorescence microscope (Jiangnan Instrument Factory Co.), after which the HaCaT were irradiated with UVB at a dose of 40 mJ/cm^2^ for 80 s, while no UVB irradiation was performed in group C. The plates were washed twice with PBS, and 2 mL of serum-free medium was added to groups C and M. OB and OBF with a volume fraction of 2.5% were added to the sample wells, respectively, and incubated in the incubator for 24 h. After 24 h, the 6-well plates were removed, washed, and 1 mL of PBS was added to them. Subsequently, the plates were photographed using a fluorescence microscope.

### ROS content determination

The effects of OBF on the ROS level in cells were verified in this experiment. 6-well plates were spread with 5 × 10^5^ cells per well. The fluorescent probe DCFH-DA was used for the detection of ROS, and the specific experimental operation was carried out according to the instruction manual.

### Enzyme-linked immunosorbent assay (ELISA)

The 6-well plates were seeded with 50 × 10^4^ cells per well, and the supernatant was collected. The plates were washed three times. Then, 200 µL of cell lysate was added to each well, and shaking was performed to facilitate cell lysis. The cells at the bottom of the plates were then collected using a cell spatula and centrifuged along with the collected supernatant. The precipitate was then discarded, and the remaining samples were used for the subsequent assay with the kit.

### qRT-PCR

Total RNA was extracted with Trizol and First Stand cDNA and manipulated for reverse transcription. Primer sequences for the desired genes were designed using PrimerExpress software. The experimental results were observed using a qRT-PCR instrument and analyzed. The reverse transcription system is shown in Table 1. The primer sequence list is shown in Table 2 (F: forward primer, R: reverse primer). The qRT-PCR reaction system is shown in Table 3.

**Table 1:** Reverse transcription system

Name of reagent	Volume (µL)
Anchored Oligo (dt) 18 Primer	1.0
2×ES reaction Mix	10.0
Total RNA	2.0
EasyScript RT/RI Enzyme Mix	1.0
Gdna Remover	1.0
Rnase-free Water	5.0

**Table 2:** Primer sequences for real-time RCR

Gene	Direction	Primer (5’-3’)
Beta-actin	F	CTGAAGCCCCACTCAATCCA
	R	GCCAAGTCAAGACGGAGGAT
TNF-α	F	TCTCCTTCCTGATCGTGGCA
	R	CAGCTTGAGGGTTTGCTACAAC
IL-4	F	TCTTTGCTGCCTCCAAGAACA
	R	GTTCCTGTCGAGCCGTTTCA
IL-6	F	TTCTCCACAAGCGCCTTC
	R	AGAGGTGAGTGGCTGTCTGT
IL-8	F	GGAGAAGTTTTTGAAGAGGGCTG
	R	ACAGACCCACACAATACATGAAG
FLG	F	TGAGGCATACCCAGAGGACT
	R	CTGTATCGCGGTGAGAGGAT
AQP3	F	CTTCTTTGACCAGGACCGGC
	R	GGGCCAGCTTCACATTCTCT
KLK-7	F	TCAGATCCTCTCGAGCCCAG
	R	CAGGTGCACGGTGTACTCAT
JAK1	F	GCATCGAGCGCACAAAGTTA
	R	GCTACTTCAGAGAAGCGTGTG
SOCS1	F	CACTTCCGCACATTCCGTTC
	R	GCATCCCAGTTAATGCTGCG
STAT1	F	GGGATACACCAGTGCACAGAA
	R	CGTACCACTGAGACATCCACA
STAT3	F	CATCCTGAAGCTGACCCAGG
	R	AGGTGAGGGACTCAAACTGC

**Table 3:** Reagents and dosage

Name of reagent	Volume (µL)
Template	1.5
Forward Primer (10 µM)	0.4
Reverse Primer (10 µM)	0.4
2×TransStart^®^ Top Green qPCR Supermix	10.0
Passive Reference Dye (50×)	0.4
Nuclease-free Water	7.3

### Safety evaluation of OBF

#### Chicken embryo chorionic allantoic membrane eye irritation test (HET-CAM)

The safety of the samples was determined by referring to the experimental procedure of Fang (Fang et al. 2023). Fertilized eggs were incubated in an incubator at 37 °C and 55% humidity for 9–10 days, and then photographs were taken to determine the location of the air chambers in the fertilized eggs. 0.9% NaCl solution and 0.1 mol/L NaOH was used as negative control and positive control in place of the samples. The egg shell of the air chamber was peeled off with forceps to reveal the white egg membrane. After wetting the membrane with NaCl solution, it was sucked out with a dropper, the inner membrane was peeled off and the corresponding samples were added dropwise to observe the situation of vascular changes and record accordingly. The eye irritation of the samples was categorized according to Table 4.

4 $$\begin{aligned} I&=\frac{(301-\text{sec}\;H)\times 5}{300}+\frac{(301-\text{sec}\;L)\times 7}{300}\\ &+\frac{(301-\text{sec}\;C)\times 9}{300}\end{aligned}$$

secH: average bleeding time.

secL: average time to vascular melting.

secC: average time to vascular clot formation.

**Table 4:** Results of evaluation for irritation score

Scoring range	Irritation classification
IS<1	No irritation
1 ≤ IS<5	Mildly irritation
5 ≤ IS<9	Moderate irritation
IS ≥ 9	Strong irritation

#### Rabbit erythrocyte hemolysis assay

Fresh rabbit blood was diluted with PBS solution at a ratio of 4:10 and centrifuged for 10 min at 1,500 g at rt. The supernatant was aspirated and the solution was centrifuged for 2–3 times with added PBS wash.

The prepared cell suspension and diluted samples of different concentrations were mixed well in a ratio of 1:3, and then incubated in a shaker at 28℃ for 60 min. After removal, they were placed in a centrifuge at 10,000 g for 1 min, OD560 was measured, and recorded as A_1_. Negative control 750 µL of PBS and 250 µL of erythrocyte suspension, ansorbance was recorded as A_2_. Positive control group 750 µL distilled water and 250µL of erythrocyte suspension, absorbance was recorded as A_3_.

5 $$\:\text{H}\text{e}\text{m}\text{o}\text{l}\text{y}\text{t}\text{i}\text{c}\:\text{r}\text{a}\text{t}\text{e}=\:\left[\right(\text{A}_{1}-\text{A}_{2})/(\text{A}_{3}-\text{A}_{2}\left)\right]\times\:100\%$$

### Data analysis

All experiments were repeated three times and each result was analyzed in three replicates, and each sample was repeated and analyzed three times. Excel and T-test were used for data processing and significance analysis. Data was expressed in mean ± standard deviation. GraphPad Prism 8 was used for analytical plotting. When *p* < 0.05, the difference was considered statistically significant.

## Results

### Physical properties of OB and OBF

**Fig. 2:**
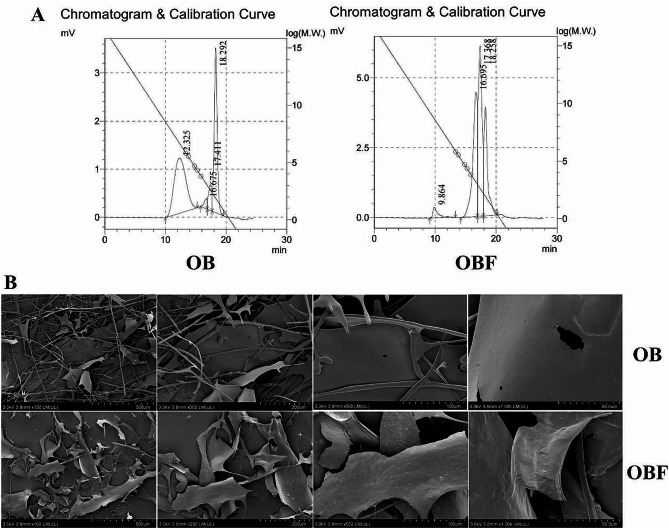
Changes in morphological and structural characteristics of oat bran before and after fermentation. (**A**): GPC chromatograms of OB and OBF. (**B**): SEM images of OB and OBF magnifications of 100, 200, 500, and 1000

The relative molecular weights of OB and OBF were determined by gel permeation chromatography (GPC), and the elution curves are shown in Fig. 2A. Molecular weight measurements (Table 5) showed that the average molecular weights of OB and OBF were 5.626633 × 10^6^ and 10.605001 × 10^6^ Da respectively. The molecular weight of OBF was higher than that of OB, which indicates that the enzymes produced during microbial fermentation help to destroy the plant cell wall, thus promoting the release of active substances. The Mn (number-average molecular weight) value of OBF is smaller than that of OB, indicating that OBF has more small molecular active structures compared to OB. Additionally, the polydispersity coefficient (Mw/Mn) of OBF is larger than that of OB, suggesting that fermented oat bran has a more complex molecular composition.

The structure and morphology of oat bran before and after fermentation were observed using scanning electron microscopy (SEM). Figure 2B shows the morphological characteristics of oat bran under different observation accuracies. From the figure, it can be seen that OBF has better structural homogeneity, and compared with OB, the surface structure of OBF is rougher and has better adhesion.

**Table 5:** Determination of OB and OBF molecular weights

	OB	OBF
Mn (Da)	165	145
Mw (Da)	5.626633 × 10^6^	1.0605001 × 10^7^
Mz (Da)	4.3279738 × 10^7^	4.93026158 × 10^8^
Mw/Mn	3.409657620 × 10^4^	7.300501671 × 10^4^
Mz/Mw	7.69194	46.48997

### Determination of OBF content

The regression equation between absorbance and glucose concentration is y = 0.003605x-0.0169. The regression equation between absorbance and pyrogallic acid concentration is y = 0.9985x + 0.0122. The regression equation between absorbance and protein standard concentration is y = 0.8868x + 0.1372. As shown in Table 6, both OB and OBF were rich in total sugars, and the total sugar content in OBF was significantly higher than that in OB. OB and OBF were also rich in total phenols and total proteins, with the total phenol content in OBF being 1.53 times higher than that in OB. The protein content in OBF was significantly higher than that in OB, being 1.46 times higher than in OB. Compared with unfermented oat bran, there was a significant increase in the content of all three bioactives after fermentation with *S. commune*.

**Table 6:** Content of total sugars, total phenols, and total proteins in OB and OBF (mean ± SD, ^b^*p* < 0.01, ^c^*p* < 0.001, compared with the OB group.)

Sample	Total Sugars (mg/g)	Total Phenols (mg/g)	Total Proteins (mg/g)
OB	120 ± 0.032	25 ± 0.001	50 ± 0.067
OBF	167 ± 0.042^c^	39 ± 0.007^b^	73 ± 0.053^c^

### Determination of OBF in vitro antioxidant activity

When more free radicals are generated in the body than its own scavenging ability can handle, it will cause damage to human health. Antioxidants can protect the body from damage caused by free radicals.

**Fig. 3:**
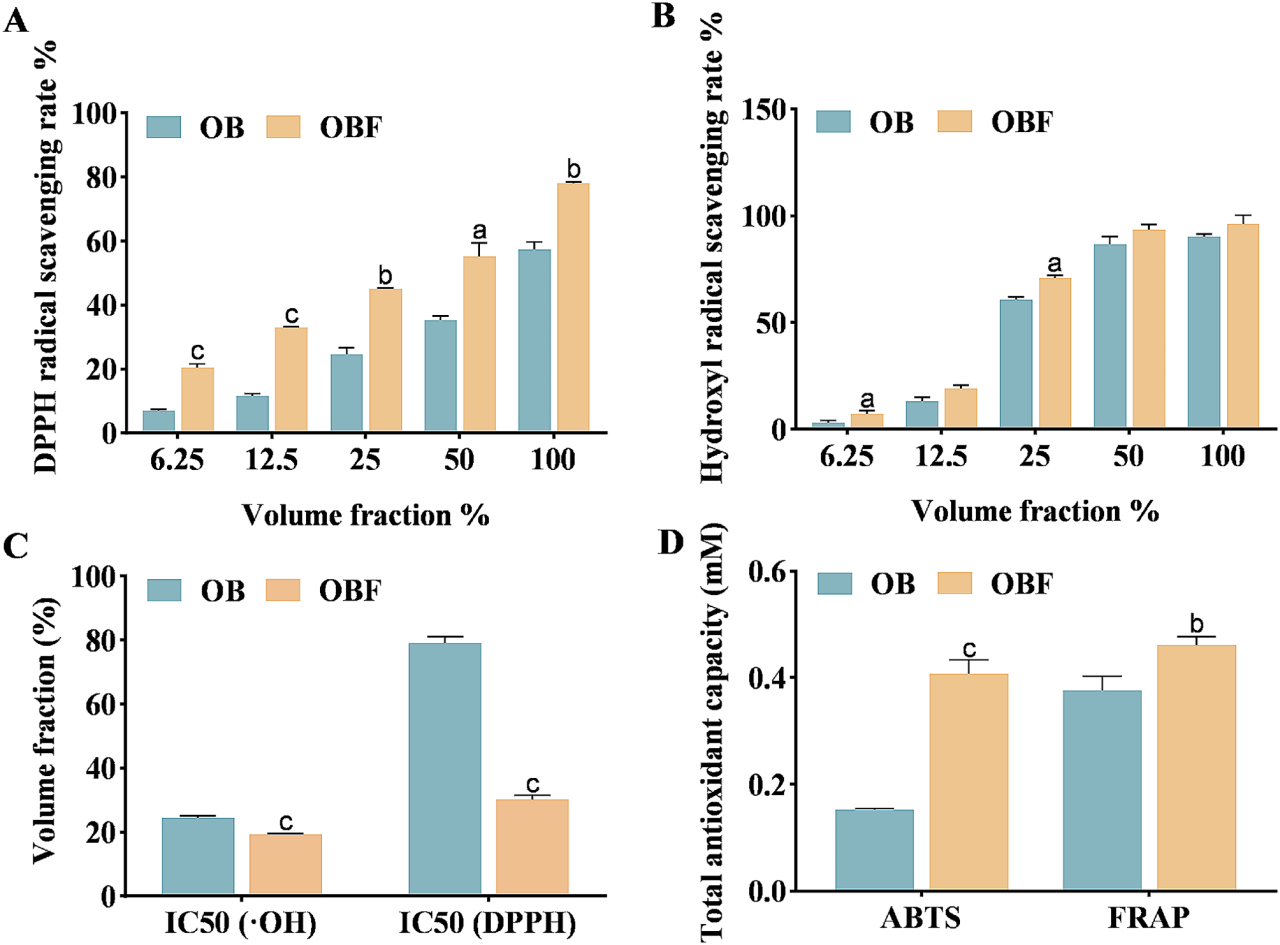
Scavenging activity of OB and OBF against oxygen radicals in vitro. (**A**): DPPH free radical scavenging capacity; (**B**): ·OH scavenging capacity; (**C**): IC50 for DPPH radicals and hydroxyl radicals. (**D**): Total antioxidant capacity. ^a^*p* < 0.05, ^b^*p* < 0.01, ^c^*p* < 0.001, compared with the OB group

The DPPH radical scavenging effect of OBF is shown in Fig. 3A. When the volume fraction of both OB and OBF samples increased, both samples exhibited stronger DPPH radical scavenging ability. When the volume fraction of OBF was 100%, the best scavenging effect on DPPH radicals was 68.01%.

The hydroxyl radical scavenging effect of OBF is shown in Fig. 3B. It can be seen that both OBF and OB had a good scavenging effect on hydroxyl radicals, with similar scavenging effects. The scavenging effect of OBF at a 50% volume fraction could exceed 90% for hydroxyl radicals, surpassing that of OB at a 100% volume fraction.

The smaller the IC50 value, the better the free radical scavenging ability of the sample. From Fig. 3C, it can be seen that OB and OBF were more effective in scavenging hydroxyl radicals and OBF was superior with a volume fraction of 19.17% scavenging 50% of hydroxyl radicals.

As can be seen in Fig. 3D, The scavenging capacity of OBF for ABTS + and the reducing capacity of Fe^2+^ were significantly higher than those of OB. Create a standard curve with absorbance on the vertical axis and Trolox concentration on the horizontal axis. The standard curve equation obtained from the ABTS radical scavenging experiment is y=-0.8717x + 0.6048. Draw a standard curve with absorbance on the vertical axis and FeSO_4_ concentration on the horizontal axis. The standard curve obtained from the FRAP iron ion reduction ability experiment is y = 0.2828x + 0.0546. The results of the ABTS method showed that OBF was 2.66 times more effective than OB in removing ABTS+, reaching 0.41 mM Trolox equivalent. The results of the FRAP method showed that the reduction capacity of OBF for Fe^2+^ was 1.23 times higher than that of OB, reaching 0.46 mM Trolox equivalent.

### Effects of OBF on cell viability

**Fig. 4:**
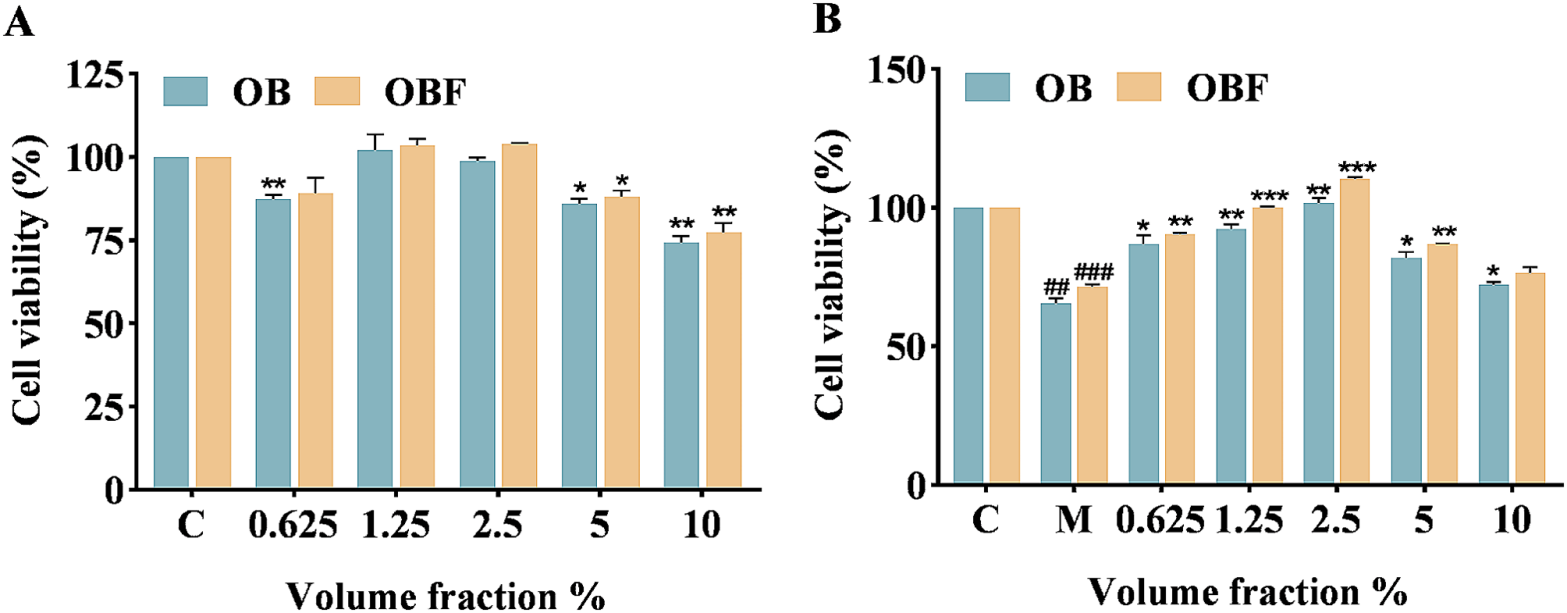
Effects of OB and OBF on cell viability. (**A**): Effect of different volume fractions of OB and OBF on cell viability. (**B**): Effect of diffferent volume fractions of OB and OBF on viability of HaCaT cells with UVB-induced damage.^*^p < 0.05,^**^p < 0.01, compared with control group; ^##^*p*<0.01, ^###^*p*<0.001 compared with control group, ^*^p < 0.05,^**^p < 0.01, ^***^p < 0.001 compared with model group

The results of the experiments without UVB irradiation (Fig. 4A) showed that the cell viability of the OBF group was significantly improved compared with that of the OB, and the cell viability was always greater than 80% when the volume fraction of OBF was in the range of 0.625-5%, which indicated that the OBF was not toxic to the cells in this volume fraction range. OBF with volume fractions of 1.25% and 2.5% had a proliferative effect on cells with cell survivals rates of 103.48% and 103.89% respectively.

It could be seen from Fig. 4B, a significant decrease in cell survival occurred in the model group after undergoing UVB damage. When the volume fraction of the sample was 2.5%, it had a significant reparative effect on the cells. The reparative effects of OB and OBF on UVB-damaged HaCaT cells decreased when the volume fraction of samples was greater than 2.5%, and the reparative effect of OBF on UVB-damaged model group was significantly better than that of OB.

By analyzing cell viability and status, we selected 2.5% as the volume fraction for both samples for subsequent cellular level and molecular level experiments.

### Effects of OBF on cell migration capacity

**Fig. 5:**
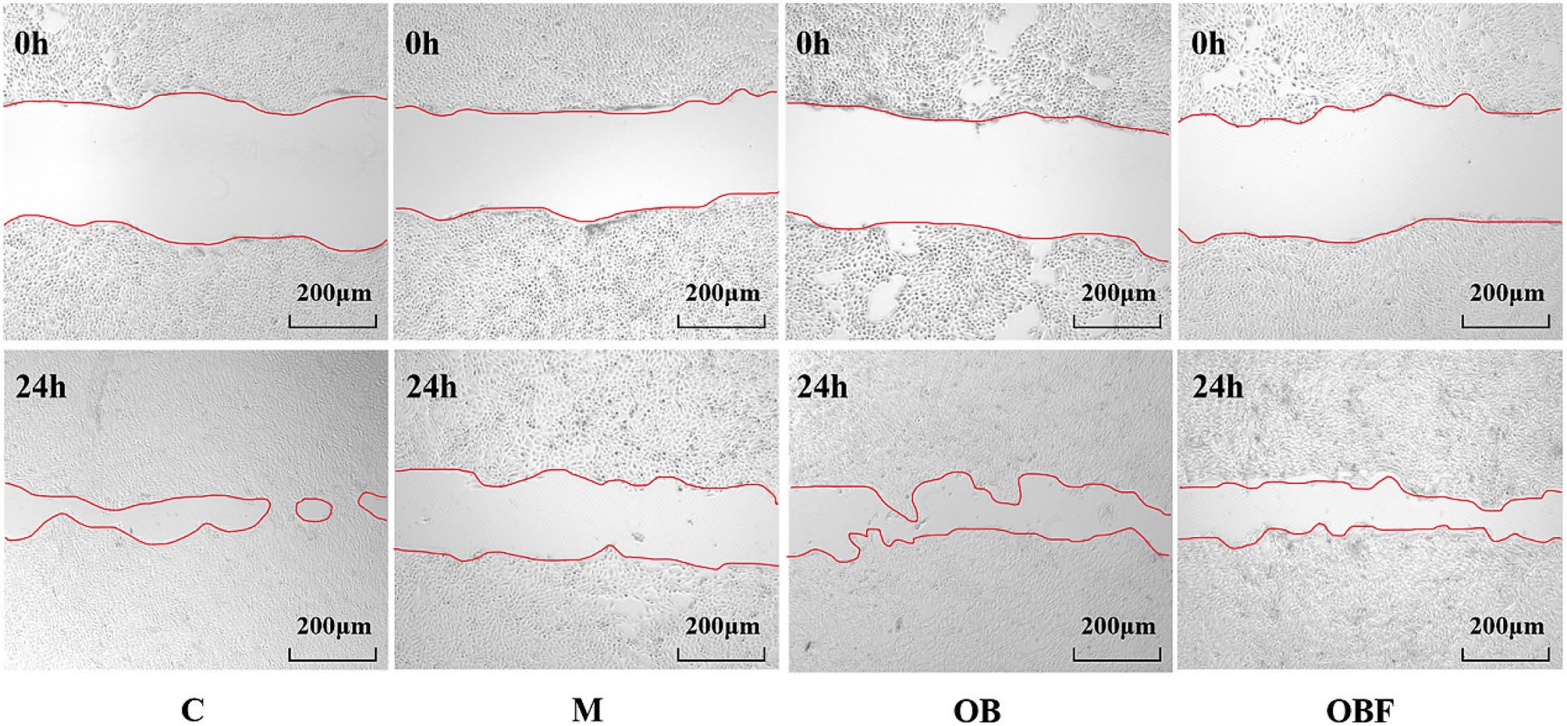
Effects of OB and OBF with a volume fraction of 2.5% on cell migration ability

Figure 5, shows the results of our measurements of cell migration capacity. After UVB irradiation, a significant decrease in cell migration ability occurred in group M, and the empty area region was significantly larger than that in group C, which was not irradiated by UVB. After UVB irradiation, the OB and OBF samples were added, and the sizes of the empty areas were calculated 24 h later. The sizes of the empty areas in the two sample groups underwent a significant decrease in comparison with that in group M, which suggests that OB and OBF at a volume fraction of 2.5% had a certain proliferative effect on cells and a certain reparative effect on damage caused to the cell migration ability by UVB irradiation. The changes in the scratched area and the calculation of the cell migration rate for the sample group are presented in Table 7.

**Table 7:** Changes in cell scratch area

	C	M	OB	OBF
Scratch Area (0 h)	4,720,963	3,616,372	3,960,228	4,070,341
Scratch Area (24 h)	860,363	2,648,581	1,682,090	1,617,335
Cell Migration Rate %	79.48%	26.76%	57.53%	60.27%

### Effects of OBF on cellular oxidative stress

Excess ROS cross cell membranes and undergo oxidative reactions with a variety of biomacromolecular substances, which in turn cause a state of oxidative stress in the body (Fernando et al. 2016). When excessive ROS are produced in the body, the ROS production-consumption balance is disrupted, and the excessive ROS promotes the release of inflammation-related factors in the body, leading to inflammation of the skin and further damaging the barrier function of the skin. Removing excess ROS is beneficial to the health of the skin. Heme oxygenase-1 (HO-1) is an enzyme with antioxidant and anti-inflammatory activities, which acts mainly by catalyzing the degradation of heme (Kim et al. 2021). NAD(P)H: quinone oxidoreductase-1 (NQO-1), a homodimeric flavoproteinase, is a representative detoxifying enzyme for phase II carcinogens (Natarajan et al. 2010).

**Fig. 6:**
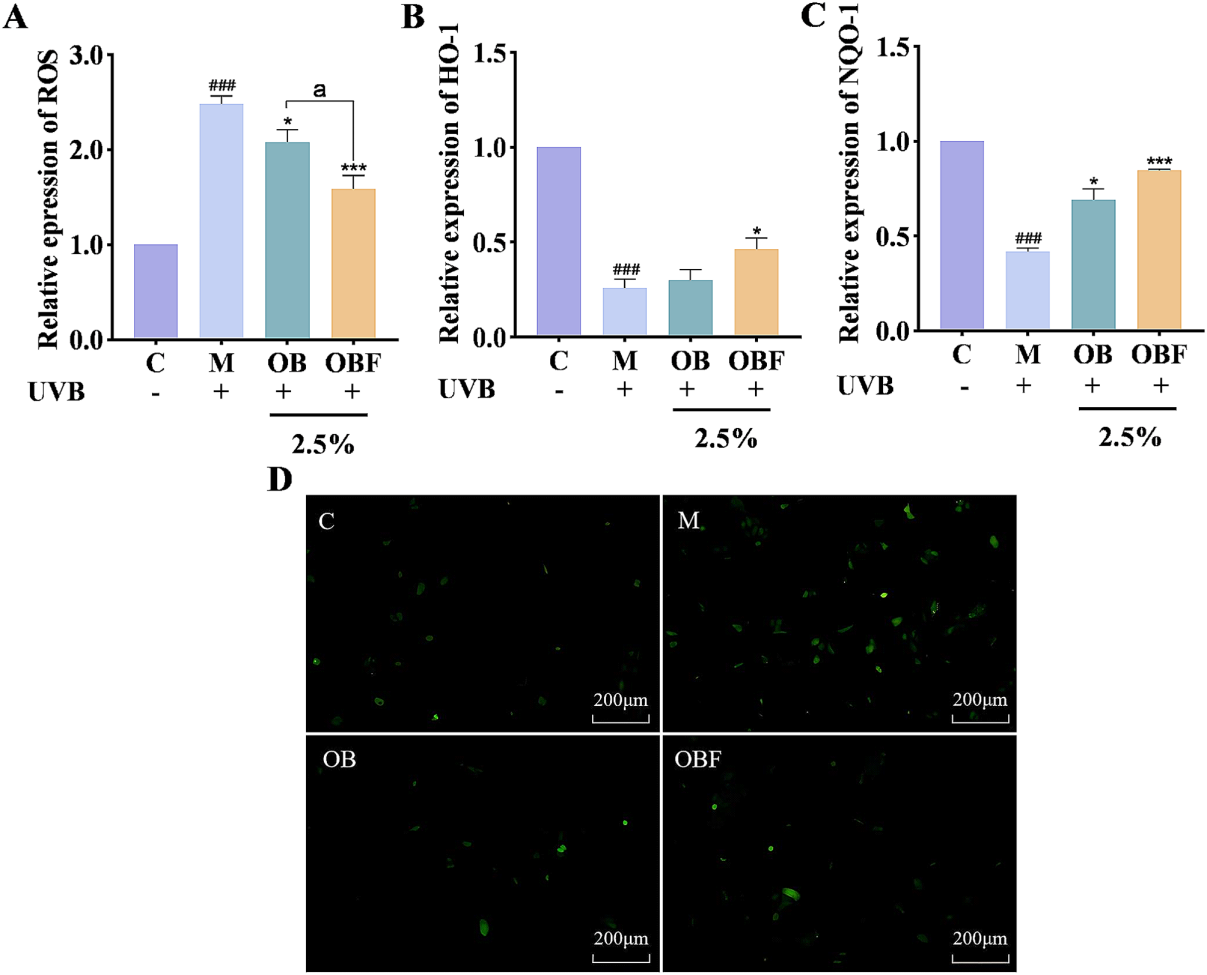
Effects of OB and OBF on ROS activity and content of antioxidant enzymes, HO-1 and NQO-1. (**A**): Cell fluorescence intensity values. (**B**): Effects of OB and OBF with volume fraction of 2.5% on HO-1 enzyme activity in HaCaT cells. (**C**): Effects of OB and OBF with volume fraction of 2.5% on NQO-1 enzyme activity in HaCaT cells. (**D**): Fluorescence intensity of ROS contained in cells. ^###^*p* < 0.001 compared with control group, ^*^p < 0.05,^**^p < 0.01, ^***^p < 0.001 compared with model group, ^a^*p* < 0.05 compared with OB group

The scavenging effect of 2.5% OBF on ROS contained in the cells is shown in Fig. 6A & D. As can be seen from the figures, the control group without UVB irradiation had less green fluorescence and lower brightness, indicating that the content of ROS in group C was lower. After UVB irradiation, the content of green fluorescence in the model group was significantly higher and brighter, indicating that the cells were damaged and the content of ROS increased after UVB irradiation. 2.5% OB and OBF were added after UVB irradiation to determine the reparative effects of the samples on HaCaT cells after UVB irradiation. The green fluorescence intensity of both the OB and OBF groups were lower than that of the model group, and the content of ROS was also reduced, which indicated that both OB and OBF had a certain reparative effect on cellular damage caused by UVB irradiation, whereas the lower green fluorescence intensity of the OBF group indicated that the effect of OBF in removing ROS was better than that of OB.

As can be seen in Fig. 6B & C, the levels of both antioxidant enzymes decreased after UVB irradiation compared to the non-UVB irradiated control group. An increase in the content of both HO-1 and NQO-1 occurred after the cells were treated with OB and OBF, and the content of both enzymes in OBF was higher than that in OB, 1.54 and 1.23 times higher than that in OB respectively.

### Effects of OBF on UVB-induced inflammatory cytokines

**Fig. 7:**
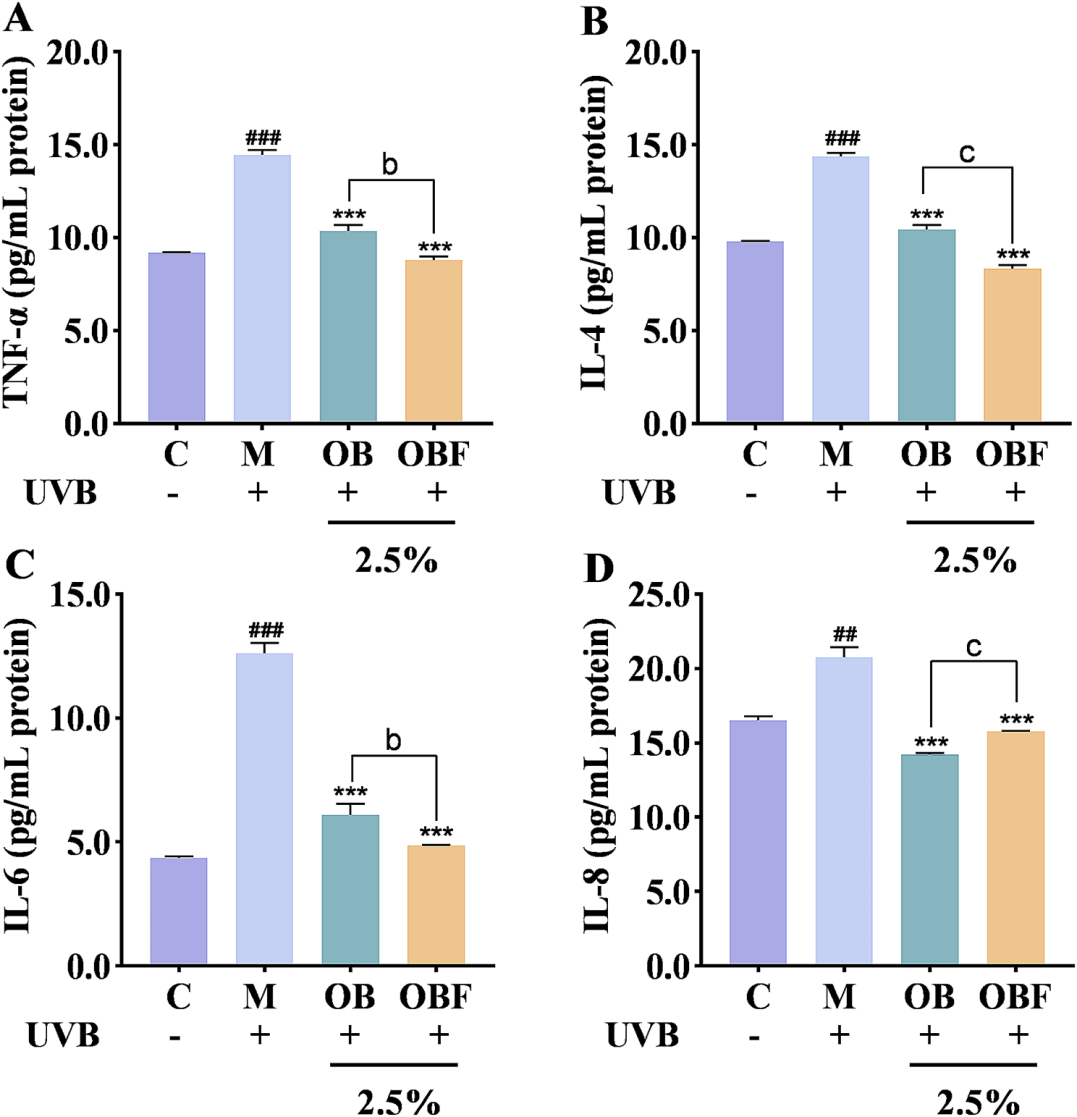
Effects of OB and OBF on inflammatory factor activity. (**A**): TNF-α inflammatory factor activity. (**B**): IL-4 inflammatory factor activity. (**C**): IL-6 inflammatory factor activity. (**D**): IL-8 inflammatory factor activity. ^###^*p* < 0.001 compared with control group, ^**^p < 0.01, ^***^p < 0.001 compared with model group, ^b^*p* < 0.01, ^c^*p* < 0.001 compared with OB group

**Fig. 8:**
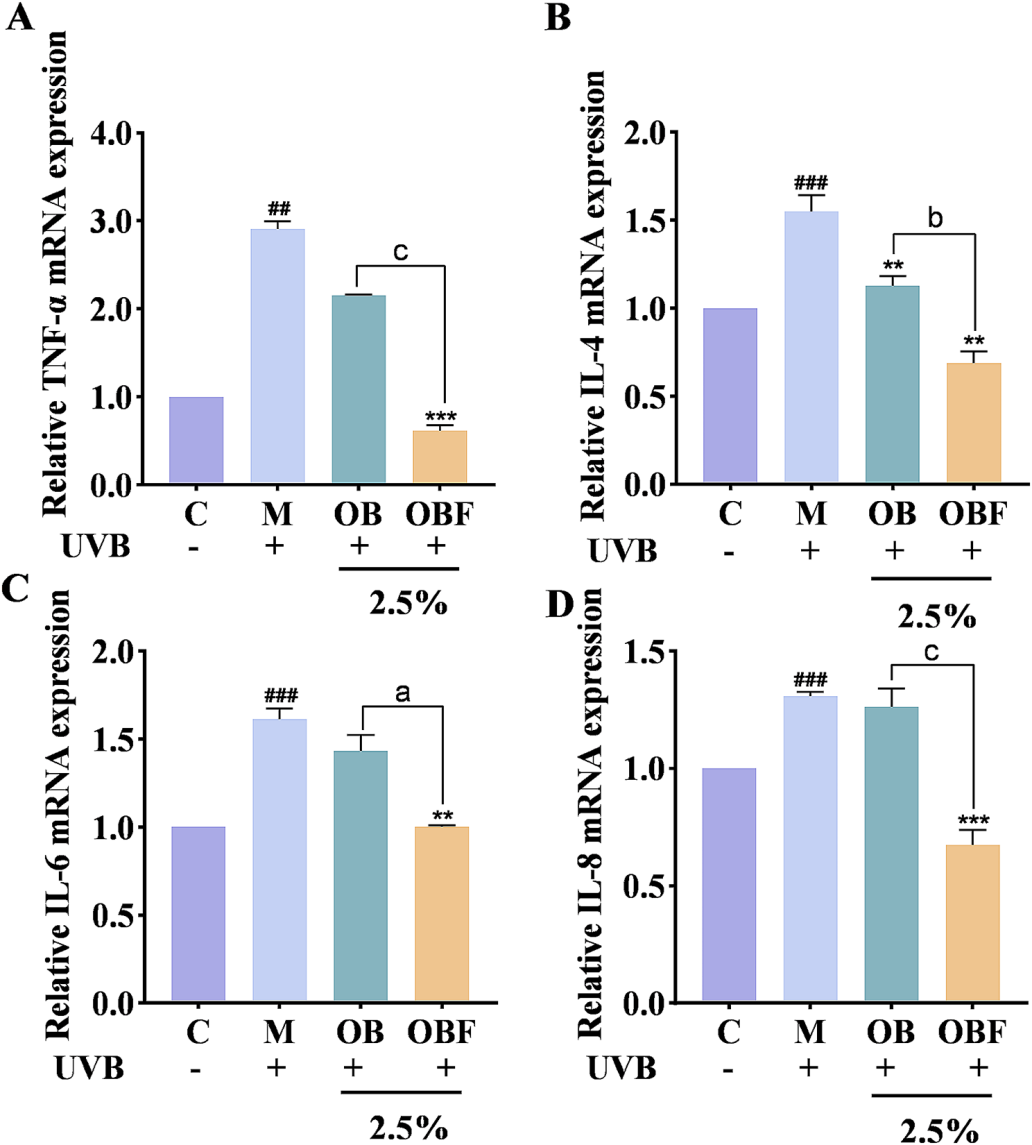
Effects of OB and OBF on inflammatory factor relative mRNA expression. (**A**) : TNF-α mRNA expression levels. (**B**): IL-4 mRNA expression levels. (**C**): IL-6 mRNA expression levels. (**D**): IL-8 mRNA expression levels. ^##^*p* < 0.01, ^###^*p* < 0.001 compared with control group, ^**^p < 0.01, ^***^p < 0.001 compared with model group, ^a^*p* < 0.05, ^b^*p* < 0.01, ^c^*p* < 0.001 compared with OB group

Exposure to excessive UVB irradiation leads to a disruption of the dynamic balance of intracellular ROS production and clearance, further contributing to a state of oxidative stress in the cells and leading to skin inflammation. TNF-α and ILs are important indicators used to evaluate whether or not an inflammatory response is occurring. The effects of OB and OBF on the content of inflammatory chemokines and their expression levels are shown in Figs. 7 and 8.

From Fig. 7, compared with the control group that did not undergo UVB irradiation, a significant increase in the content of all four inflammatory factors can be seen in the model group.

From Fig. 8, after UVB irradiation, the relative mRNA expression of all four inflammatory factors underwent a significant increase, OB and OBF with a volume fraction of 2.5% had an inhibitory effect on the mRNA expression of all four inflammatory factors, and the inhibitory ability of OBF on the release of inflammatory factors was superior to that of OB.

### Effects of OBF on UVB-induced skin barrier-related factor content

With the assistance of FLG monomer in the connection, keratin fibers regularly gather in the outermost layer of the epidermis, forming a strong physical barrier, thus preventing water loss and the entering of external irritants (Hoyer and Rehbinder 2022). AQP3 is expressed predominantly in the plasma membrane of epidermal keratinocytes and transports water and glycerol (Schrader et al. 2012). In immature skin, which lacks an effective skin barrier, AQP3 plays a role in epidermal hydration and transdermal water transport (Agren et al. 2010). Initially kallikrein-7 (KLK-7) was thought to be an enzyme involved in the degradation of intercellular cohesive structures in the stratified squamous epithelial stratum corneum that catalyzes the degradation of the skin’s outermost bridging granules, resulting in the detachment of cells from the surface of the skin (Kumar et al. 2020).

**Fig. 9:**
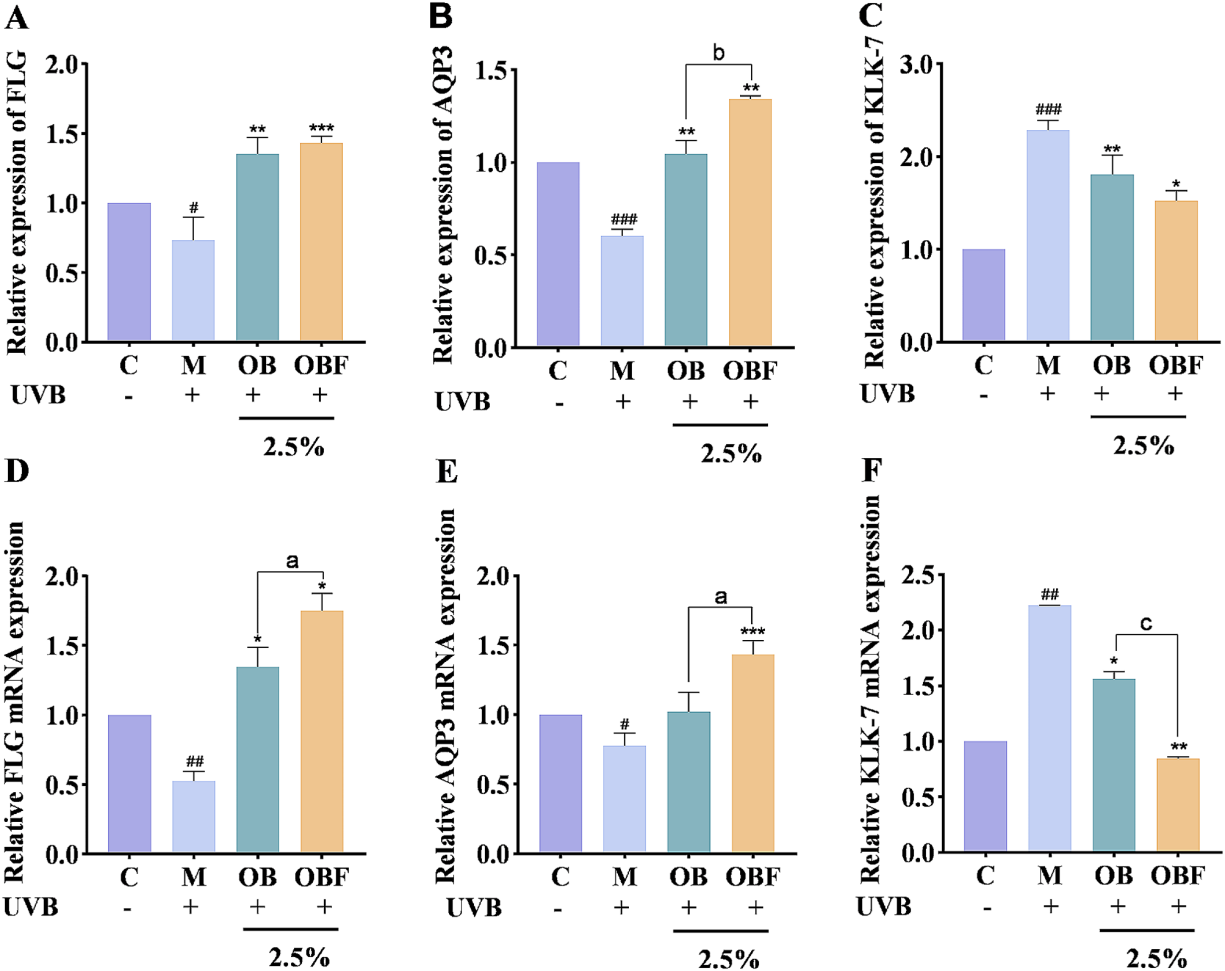
Effects of OB and OBF on the viability of barrier-associated factors and their mRNA expression. (**A**): FLG factor activity. (**B**): AQP3 factor activity. (**C**): KLK-7 factor activity. (**D**): FLG mRNA expression level. (**E**): AQP3 mRNA expression level. (**F**): KLK-7 mRNA expression level. ^#^*p*< 0.05, ^##^*p* < 0.01, ^###^*p* < 0.001 compared with control group, ^*^p < 0.05,^**^p < 0.01, ^***^p < 0.001 compared with model group, ^a^*p* < 0.05, ^b^*p* < 0.01, ^c^*p* < 0.001 compared with OB group

In Fig. 9, the amount of both FLG and AQP3 proteins in the model group without samples after UVB irradiation was significantly reduced, and the enzyme activity of KLK-7 was significantly increased. After irradiation with UVB and treatment with OB and OBF at a volume fraction of 2.5%, the relative expression of FLG was significantly increased compared with that of the model group, which were 2.55 and 3.32 times higher than that of the model group respectively. After treating the cells with OB and OBF, the relative expression of AQP3 was higher than that of the model group and even higher than that of the control group which was not irradiated by UVB, and the effect of OBF was better than that of OB. After treating the cells with OB and OBF, the relative expression of KLK-7 was significantly reduced compared with that of the model group, which were 0.70 and 0.38 times that of the model group respectively, and the effect of OBF was better than that of OB.

### OBF can act on the JAK/STAT pathway

**Fig. 10:**
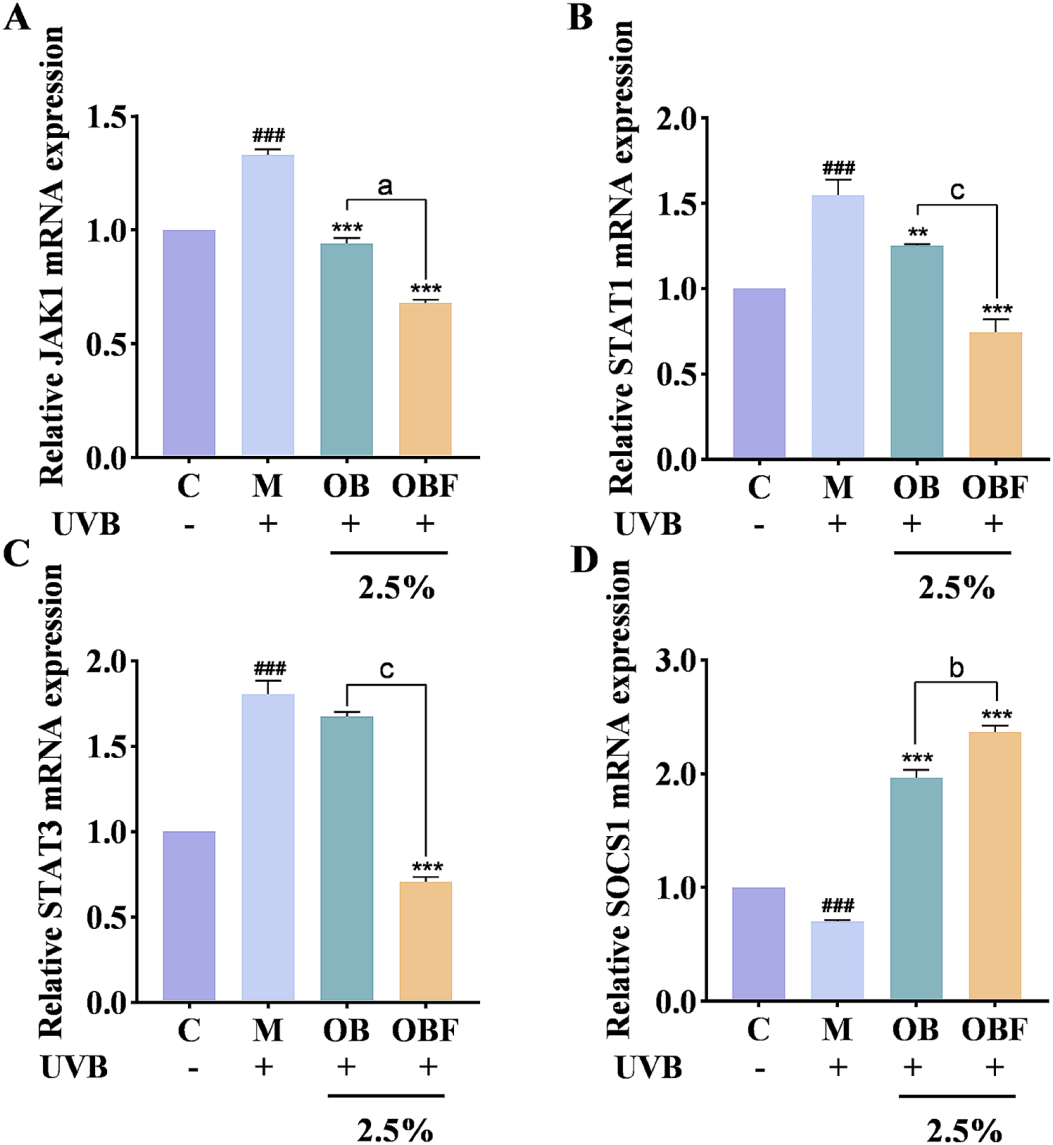
Effects of OB and OBF on JAK/STAT pathway gene expression in HaCaT cells (**A**–**D**): effects of OB and OBF at 2.5% concentration on JAK1, STAT1, STAT3 and SOCS1. ^###^*p*<0.001 compared with control group, ^**^p < 0.01, ^***^p < 0.001 compared with model group, ^a^*p* < 0.05, ^b^*p* < 0.01, ^c^*p* < 0.001 compared with OB group

In Fig. 10, after UVB irradiation, the relative expression of JAK1, STAT1 and STAT3 mRNA were significantly increased, and the relative expression of SOCS1 mRNA was significantly decreased. After treating the cells with OB and OBF at a volume fraction of 2.5%, a significant decrease in the transcript levels of JAK1, STAT1 and STAT3, and a significant increase in the transcript level of SOCS1 occurred, and the effect of OBF was superior to that of OB. This suggests that OBF may be able to exert a reparative effect on UVB-induced skin inflammation by inhibiting the activation of the JAK/STAT pathway in HaCaT cells.

### Safety of OBF

**Fig. 11:**
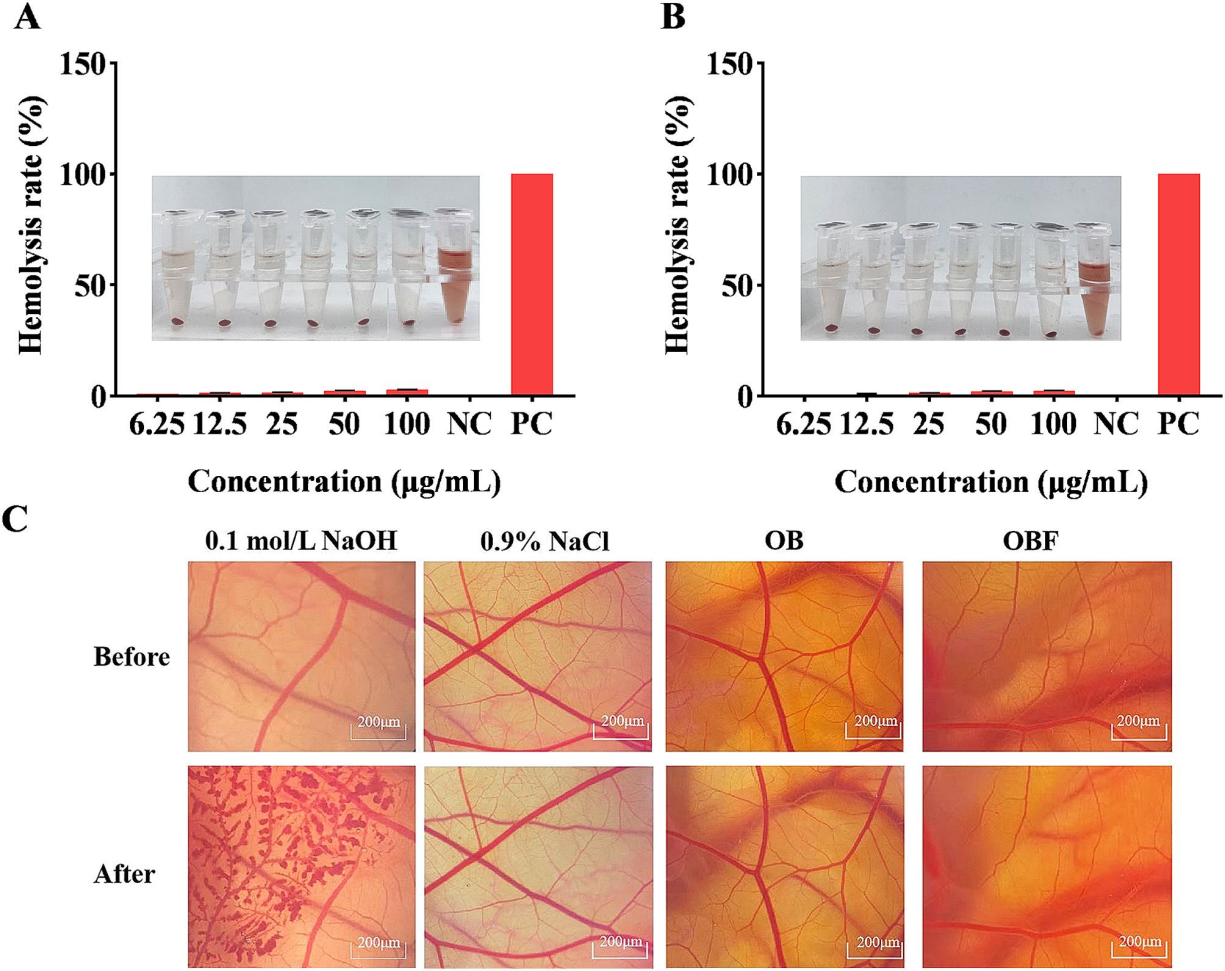
Safety of OBF and OB. (**A**) : Effect of OB on rabbit erythrocyte haemolysis; (**B**): Effect of OBF on rabbit erythrocyte haemolysis; (**C**): Effects of OB and OBF on CAM vascular

Figure 11A & B show the effects of different concentrations of OB and OBF on the hemolytic capacity of rabbit erythrocytes. The experimental results showed that the effect of different mass concentrations of the samples on the hemolysis of rabbit erythrocytes were relatively small, which indicated that OB and OBF had high safety profiles.

The Fig. 11C shows the stimulating effect on blood vessels. The stimulation score of the positive control group (NaOH) was calculated to be 18.45, with obvious hemolysis and strong stimulation. The negative control group (NaCl) had a stimulation score of 0.03, and there was no hemolysis in the blood vessels. The stimulation scores of OB and OBF were 0.08 and 0.07 respectively, and there was no hemolysis in the blood vessels, indicating that OB and OBF did not stimulate the eyes.

## Discussion

A small amount of UVB radiation is necessary for the body, but excessive exposure can lead to the overproduction of ROS in the human body. This disrupts the delicate balance of the body’s antioxidant defense system, causing oxidative stress in the cells. Ultimately, this can result in skin inflammation, damage to the skin barrier, and the impairment of the skin’s defense function. When the skin is inflamed, the secretion of pro-inflammatory factors linked to skin inflammation, such as IL-6, IL-8 and TNF-α, increases. This leads to redness, itchiness, and flakiness appearing on the skin’s surface.

Scavenging excessive intracellular ROS and inhibiting the release of pro-inflammatory factors are effective strategies for alleviating skin inflammation caused by UVB irradiation. HO-1 and NQO-1 are two enzymes with antioxidant effects. Both enzymes can alleviate the oxidative stress in cells by scavenging excessive ROS. ROS is an effective inducer of the JAK/STAT pathway, which is closely related to many immune and allergic diseases. UVB irradiation promotes the activation of JAKs phosphorylation, and STATs are the downstream genes of JAKs. After the phosphorylation of JAKs, the activated STATs are translocated to the cell nucleus to regulate multiple target genes.

Oat bran contains a large number of polyphenolic compounds, sugar compounds, proteins, and other biologically active substances that have various effects, such as antioxidation and anti-inflammation. *Schizophyllum commune* is a wild fungus that contains a potent active cellulase enzyme with antioxidant, anti-tumor, and anti-aging properties. It has been shown that the oat bran fermented by *Schizophyllum commune* can repair UVA/H_2_O_2_-induced oxidative stress damage through the PI3K-AKT pathway. However, few studies have been conducted on the reparative effects of oat bran fermentation broth fermented by *Schizophyllum commune* on skin inflammation caused by UVB irradiation through the JAK/STAT pathway.

We established a HaCaT cell model of UVB-induced injury and explored the reparative effects of oat bran fermented by *Schizophyllum commune* on UVB-induced skin inflammation at the in vitro, cellular, and molecular levels. This study provides a theoretical basis and supporting data for the application of oat bran fermentation broth fermented by *Schizophyllum commune*. The physical properties of OBF were investigated using GPC and SEM. The experimental results indicated that the molecular weight of OBF was higher than that of OB. This difference could be attributed to the enzymes produced during microbial fermentation, which break down the plant cell wall and facilitate the release of active substances like macromolecular polysaccharides. Additionally, OBF exhibited better structural homogeneity, a rougher surface, and a superior adherence effect. Significant increases in total phenols, total sugars, and total proteins were observed in oat bran, which could be attributed to a certain degree of enrichment of bioactive substances during the fermentation process. The experimental results of the four in vitro antioxidant assays showed that both OB and OBF exhibited superior in vitro antioxidant capacity. Additionally, OBF demonstrated better scavenging ability for free radicals compared to OB. The impact of OBF on the viability of HaCaT cells was assessed using the CCK8 method. Additionally, the influence of OBF on cell migration ability was investigated through a cell scratch assay. Subsequently, OBF at a volume fraction of 2.5% was chosen for further experiments. Compared with OB, OBF exhibited a superior effect in scavenging ROS. Furthermore, the relative expression levels of HO-1 and NQO-1 increased after fermentation. After UVB irradiation, the content and transcript levels of four inflammatory factors, TNA-α, IL-4, IL-6, and IL-8, increased in the cells and could be effectively alleviated with OBF. By measuring the content and transcript levels of genes related to the skin barrier, namely FLG, AQP3, and KLK-7, it was found that treatment with OBF had a reparative effect on barrier damage caused by UVB irradiation. The results at the molecular level suggest that OBF may be able to exert a reparative effect on skin photoinflammation caused by UVB-induced damage through the JAK/STAT pathway. The safety of OBF was assessed using the chick embryo assay and erythrocyte hemolysis assay, demonstrating a high safety profile for OBF.

Although our study initially demonstrated that the content of active substances and antioxidant capacity of oat bran were enhanced after fermentation by *Schizophyllum commune*, we focused our study on the fermentation broth and did not investigate the specific substances in the fermentation broth. In order to better utilize the oat bran fermentation broth fermented by *Schizophyllum commune*, more experiments at the molecular level should be carried out to further explore the specific substances and targets of its action.

## Conclusion

In this study, we investigated the alterations in the physical properties of oat bran following fermentation by *Schizophyllum commune.* Subsequent experiments were carried out at the biochemical, cellular, and molecular levels. The results of the physical property measurements showed that the molecular weight of oat bran increased, and structural homogeneity was improved after *S. commune* fermentation. By analyzing the samples for active substance content, free radical scavenging capacity, and inflammatory repair capacity, we found that fermented oat bran had higher active substance content, better antioxidant capacity, and superior inflammatory repair capacity. Molecular level experiments also demonstrated that fermented oat bran had a superior reparative capacity on UVB damage associated with the JAK/STAT pathway. The experimental results of the safety assay showed that OBF had a high safety profile.

## Data Availability

The data that support the findings of this study are available on request from the corresponding author Dongdong Wang, upon reasonable request.
